# Nasal exudate for diagnosis of stroke: fundamental studies through iron fractionation, total iron, and targeted protein determinations

**DOI:** 10.1007/s00216-024-05469-5

**Published:** 2024-08-17

**Authors:** Marta Marina-Latorre, Lara Lobo, Carmen García-Cabo, Lorena Benavente-Fernández, Sergio Calleja-Puerta, M. Teresa Fernández-Abedul, Héctor González-Iglesias, Rosario Pereiro

**Affiliations:** 1 Department of Physical and Analytical Chemistry, University of Oviedo, Julián Clavería 8, 33006 Oviedo, Spain; 2 Department of Neurology, Central University Hospital of Asturias, Av. Roma s/n, 33011 Oviedo, Spain; 3 Department of Technology and Biotechnology of Dairy Products, Instituto de Productos Lácteos de Asturias, Consejo Superior de Investigaciones Científicas, Villaviciosa, Spain

**Keywords:** Stroke, Nasal exudate, Isotope dilution analysis, Elemental analysis, HPLC-ICP-MS, Size exclusion chromatography

## Abstract

During the last years, there has been an increasing research interest in the analysis of biological fluids requiring non-invasive sampling for biomedical and clinical applications. In this work, we have focused on the nasal exudate with the aim of investigating the potential use of this fluid to know the role of iron in stroke and also for diagnosis. Potential differences in the nasal exudate, collected in swabs, from diagnosed hemorrhagic stroke, ischemic stroke, and control groups were investigated with regard to total iron by inductively coupled plasma-mass spectrometry, iron fractionation studies by size exclusion chromatography together with post-column isotope dilution analysis, and four proteins containing iron (ferritin, transferrin, lactoferrin, and ferroportin) with ELISA kits. All these analyses represent an analytical challenge, considering the rather limited amount of sample (10–40 mg) available, being the nasal exudate extracted from the swab with 300 µL 10 mM Tris/HCl, pH = 7.4. Studies to obtain reliable analytical information, such as the blank contribution of the sampling step, evaluation of the extraction efficiency of the nasal exudate from the swab, and normalization strategies for data treatment, have been carried out. Results showed that despite the limited number of investigated samples, fractionation studies as well as the concentrations of ferritin and ferroportin obtained with ELISA kits showed a differential behavior between the different cohorts.

**Supplementary Information:**

The online version contains supplementary material available at 10.1007/s00216-024-05469-5.

## Introduction

Stroke is the first cause of death in women and the second in men worldwide. It is also a main cause of long-term disability; in fact, just at about 50% of survivors are left without sequelae [1]. Stroke prognosis strongly depends on the affected area of the brain and the time elapsed between the first symptoms and the therapeutic management [2, 3]. There are two types of stroke, ischemic and hemorrhagic, both producing a decrease in the blood flow to the brain. Ischemic stroke occurs when the blood supply to a part of the brain is blocked or reduced; this prevents brain tissue from getting oxygen and nutrients and brain cells begin to die in minutes. On the other hand, hemorrhagic stroke occurs when a blood vessel in the brain leaks and causes bleeding in the brain; this blood increases pressure on brain cells and damages them. Diagnosis is conventionally done with imaging techniques such as computed tomography scan of the head and magnetic resonance imaging. Despite that much research has been devoted to study blood markers associated with this pathology, poor outcome has been obtained to date, probably due to the protection offered by the blood–brain barrier [4, 5]. Research has been also focused on the analysis of cerebrospinal fluid as a source of information from the brain [6]; however, the need for an invasive sampling makes the use of this fluid difficult to generalize [7].

In recent years, there has been an increased interest in the analysis of biological fluids which do not require of invasive sampling for biomedical and clinical applications [8, 9]. For instance, nasopharyngeal swab test is sadly well known in the frame of the respiratory infectious disease caused by the SARS-CoV-2 virus. Other examples of applications related to nostril fluids focus on investigating expression changes in the lung airway transcriptome in asthmatic patients [10] or in the context of allergic rhinitis [11]. More recently, nasal exudate has been explored as an option to obtain direct information from the brain [3, 12, 13] justified by the existence of a lymphatic drainage from the brain to the nasal mucosa.

Iron in the body is required for DNA synthesis, mitochondrial respiration, biosynthesis of neurotransmitters, axonal growth, and receptor-mediated postsynaptic signal transduction [14]. In the brain, accumulation of this element in certain areas naturally occurs with aging, but can be also observed as a consequence of genetic mutations and inflammation to the point of altering iron metabolism leading to different disorders [15], including Alzheimer’s [16, 17], Parkinson’s [18], and stroke [19, 20], among others [21]. Iron is mainly associated with proteins and is known to be toxic in free form. Therefore, iron accumulation must be linked with proteins involved in the regulation metabolism of this element; as an example, in case of stroke, it has been proved that binding affinity of transferrin for iron decreases after ischemia [22].

As stated above, nowadays, nasal exudate is considered a non-invasive source of biomedical attractiveness, being of high interest to evaluate the extent of providing information from the brain. In fact, total levels of iron measured in this fluid have proved to provide statistical differences between individuals diagnosed with ischemic or hemorrhagic stroke [12]. Nevertheless, basic studies are required to obtain reliable analytical information, such as the blank contribution of the sampling step and the potential need for signal normalization procedures.

Taking into account the interest to deepen into the potential use of nasal exudate as a source of stroke information of the brain and the main role of iron metabolism in relation to this pathology, the present work focuses on investigating potential differences in terms of iron levels: iron bound to proteins and total iron measured in nasal exudate in three cohorts of human subjects (hemorrhagic stroke, ischemic stroke, and control groups) by means of inductively coupled plasma-mass spectrometry (ICP-MS), isotopic dilution (ID), and size exclusion chromatography (SEC)-ICP-MS [23]. In addition, concentrations of four iron-binding proteins (ferritin, transferrin, lactoferrin, and ferroportin) have been determined in nasal exudate by means of commercial ELISA kits. It must be highlighted that in this work different types of analysis are carried out (elemental, SEC–ICP–MS, and ELISA). Therefore, considering the low volumes available for this type of sample, the achievement of the general goal represents a challenge in modern analytical chemistry.

## Experimental

### Sample collection and preparation

Nasal exudate was collected at Central University Hospital of Asturias (ischemic and hemorrhagic patients) and at the Faculty of Chemistry (8 control individuals) from both nostrils using a disposable swab (Biocomma Ltd, China) and directly frozen at − 80 °C inside a non-sterile graduated tube (CTGP-015–100, Labbox, Spain). This research was conducted in accordance with the Declaration of Helsinki ethical principles for medical research involving human subjects and was approved by the Hospital Universitario Central de Asturias Ethical Committee (approval number 198/16). Patients from hemorrhagic (*n* = 8) and ischemic stroke (*n* = 7) were recruited within 48 h of symptom onset. At admission, patients underwent neurological examination assessed by the National Institutes of Health Stroke Scale (NIHSS) and brain computed tomography scan. Table S1 in the Electronic Supplementary Material (ESM) collects information regarding sex and age of stroke patients and control individuals, as well as nasal exudate weight for each sample, measured by weighing the swab before and after sample collection.

**Fig. 1:**
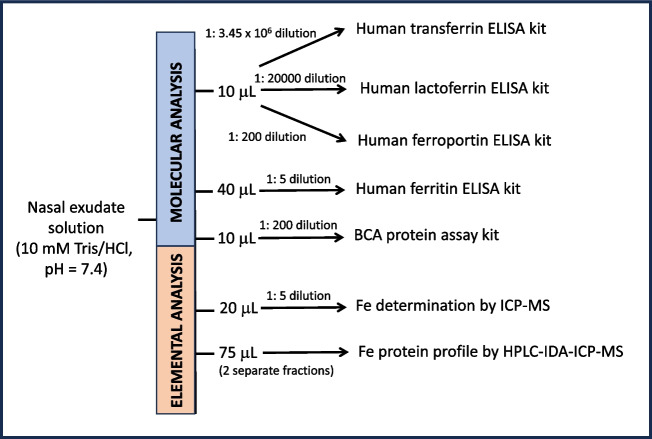
Scheme of the experimental design collecting how the nasal exudate solution available after extraction from the swab was distributed along the different experiments

For analysis, nasal exudate was unfrozen and extracted from the swab with 300 µL of 10 mM Tris/HCl, pH = 7.4 as follows: (i) the swab was removed from the storing tube and introduced into a pre-cleaned 500-µL Eppendorf tube; (ii) 300 µL 10 mM Tris/HCl, pH = 7.4, was used to rinse the tube where the swab was previously stored; such volume was then added to the Eppendorf tube; (iii) at this point, the swab was stirred at about 1 min inside the solution, squeezed against the walls of the tube and discarded; (iv) once extracted nasal exudate (in 300 µL), the solution was passed through a 0.22-µm syringe low binding protein filter (ref. SLGV013SL, Millipore, Ireland) made of PVDF membrane. Those filters are registered by the FDA and can be used as medical device according to the supplier. The solution with this filtered nasal exudate is further referred to as “nasal exudate solution.” After extraction and filtration, nasal exudate was aliquoted according to the scheme collected in Fig. 1. Each of the fractions was then frozen at − 80 °C until analysis.

### Total Fe and Fe fractionation analysis

Elemental analysis of total iron was performed with an ICP-MS (Agilent 7900, USA) equipped with a collision/reaction cell using helium gas (4.0 mL min^−1^ flow rate). The system CytoNeb Single Cell Nebulizer combined with a total consumption CytoSpray chamber (Elemental Scientific Inc, Spain) was employed for sample introduction at an aspiration rate of 12 µL min^−1^, offering 3.5 µg L^−1^ Fe as the limit of quantification. Quantitative analysis was accomplished by means of external calibration (up to 30 µg L^−1^ Fe standards were prepared) using Ga as internal standard (10 µg L^−1^) in 0.28 M HNO_3_ (TraceMetal™ Grade, Fisher Scientific, Spain) that was prepared from standard stock solutions with concentrations of 1000 µg L^−1^ (Merck, Spain). 50 µL of a 1:5 dilution, prepared directly from the nasal exudate solution and 0.28 M HNO_3_, was used for elemental determination of iron. This dilution was prepared in pre-cleaned (using 0.28 M HNO_3_) Eppendorf tubes. Other experimental operating parameters carried out related to ICP-MS analysis are detailed in Table 1. Procedural blanks following the same steps, but using a swab without nasal exudate, were also prepared. The analysis of total iron in all samples was carried out in a single session.

**Table 1:** Operating parameters employed in the ICP-MS measurements

Plasma conditions	Reaction/collision cell parameters	Data acquisition parameters
RF power: 1550 W	He gas	Monitored isotopes: ^54^Fe, ^56^Fe, ^57^Fe, ^58^Fe, and ^69^ Ga
Auxiliary gas: 0.9 L min^−1^	Flow rate: 4.0 mL min^−1^	Points per peak: 3
Plasma gas: 15 L min^−1^		Integration time per mass: 100 µs
Make-up gas: 0.47 L min^−1^		Analysis time per chromatogram: 40 min
Nebulizer gas: 0.7 L min^−1^		

**Fig. 2:**
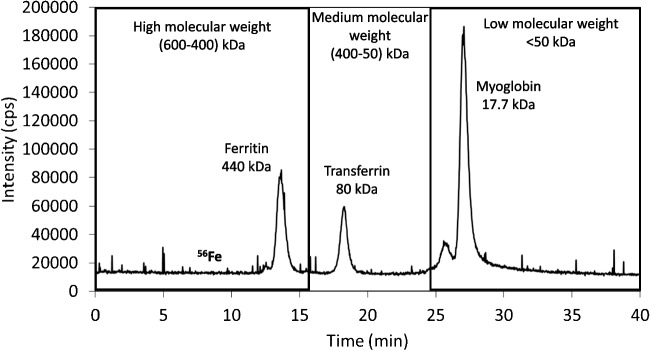
Chromatogram obtained for column calibration by SEC–ICP–MS monitoring ^56^Fe for ferritin, transferrin, and myoglobin

The chromatographic system consisted of a Thermo Finnigan Surveyor LC pump plus (Thermo Scientific, Spain) at a flow rate of 0.6 mL min^−1^ using a buffer solution of 10 mM Tris HCl (pH = 7.4) and a Rheodyne Model 7125 (Cotati, CA, USA) injection valve fitted with a 50-µL loop. A Superdex 200 Increase 10/300GL size exclusion column (I.D. 30 cm × 10 mm, 13 μm avg. part. size) from Cytiva (USA) was employed. A scavenger column (25 × 0.5 mm id), packed with Kelex-100 [24] (Schering, Germany), was used to remove metal ions present in the mobile phase. A 10 ng mL^−1 54^Fe (96.80% abundance of ^54^Fe)-enriched standard solution was continuously introduced (at 0.10 mL min^−1^) with a peristaltic pump (Miniplus 3, Gilson, Spain) through a T piece. Elemental detection was performed with the Agilent 7900 ICP-MS, fitted with a conventional Meinhard nebulizer and with a double-pass spray chamber, Peltier cooled to 2 °C. Quantification of iron-binding fractions in the nasal exudate was carried out by on-line post-column ID analysis, as described elsewhere [25], thus converting qualitative chromatograms into mass flow chromatograms. Mass of iron (ng) was calculated by integration of each chromatographic peak. Column calibration was performed by using three iron proteins, each of them eluting in a different molecular weight region: ferritin (440 kDa) in the high, transferrin (80 kDa) in the medium, and myoglobin (17.7 kDa) in the low molecular weight region. An example of a chromatogram obtained for column calibration by SEC–ICP–MS under the selected operation conditions is collected in Fig. 2. OriginPro 2024 was used both for integration of the chromatographic peaks and for statistical analysis. Unless stated otherwise, statistical differences were calculated at 95% confidence level.

### Targeted quantification of transferrin, ferritin, lactoferrin, and ferroportin by immunoassays

Quantification of selected iron-binding proteins was performed with commercial ELISA kits following the instructions of the manufacturer. Commercially available sandwich-type ELISA kits from Abcam (Cambridge, UK) were used for the quantification of transferrin (ab288174), ferritin (ab108837), and lactoferrin (ab200015). Ferroportin (MBS450431) was determined by a commercial sandwich-type ELISA kit from MybioSource (USA). Total protein concentration in nasal exudate was determined using the commercial Pierce BCA Protein Assay Kit (Thermoscientific, Spain). All absorbance measurements were monitored with the spectrophotometer PerkinElmer 2030 Multilabel Reader VICTORTM X5 (Waltham, MA, USA).

## Results and discussion

### Procedural blanks, efficiency of the extraction process, and contamination issues

#### Procedural blanks

Taking into account the limited amount of nasal exudate collected in the swab (10–40 mg), it is critical to evaluate procedural blanks in terms of iron contamination as well as whether the protein profile could be affected by possible exogenous iron during the sampling and extraction process, due to the presence of iron in the swab cotton, in the tubes used for sample extraction, or in the chemicals. Therefore, potential contamination arising from the extraction process was considered, both in terms of total iron concentration and the iron-protein profile. For such purpose, 300 µL of 10 mM Tris/HCl, pH = 7.4 (previously passed through a home-made minicolumn packed with Kelex-100 resin), was pipetted into a 500-µL Eppendorf tube and, subsequently, the swab without sample was introduced in the tube to proceed with the protocol described above. Total concentration of iron was determined in the extract for three independent swabs. Procedural blanks showed total iron levels of 8.6 ± 0.1 ng mL^−1^ (*n* = 3 procedural replicates) whereas iron concentration measured in sole 0.28 M HNO_3_ by conventional nebulization was in all cases below 1 ng mL^−1^.

In addition, the procedural blank extracts were analyzed by SEC-ID-ICP-MS. However, no peaks were observed in the iron speciation chromatogram after blank injection, and so, despite the increased total level of iron, no contribution in terms of iron species was detected above the detection limit of the methodology.

#### Efficiency of nasal exudate extraction

**Fig. 3:**
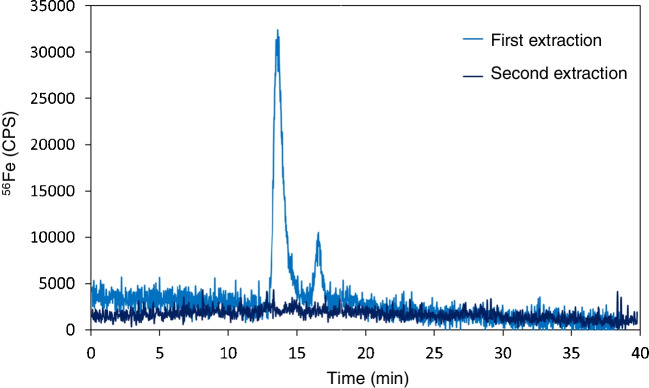
Chromatogram obtained by monitoring ^56^Fe signal after two subsequent extractions of a nasal exudate sample using the same swab

Next, the efficiency of the nasal exudate extraction process from the swab was evaluated. Nasal exudate of a control individual was collected and directly frozen at − 80 °C for at least 24 h (same procedure as for stroke samples collected at the Hospital). Nasal exudate was unfrozen and then extracted from the swab using 300 µL of 10 mM Tris/HCl, pH = 7.4. A second consecutive extraction, using additional 300 µL of 10 mM Tris/HCl, pH = 7.4, was tackled in the same swab and collected separately. Iron fractionation by SEC–ICP–MS was performed in the two extracted fractions (after filtration through 0.22-µm filters) to check whether the nasal exudate was completely removed from the swab in a single step. Figure 3 shows the chromatogram obtained after the subsequent extractions of the same swab. It can be seen that two peaks are eluted in the chromatogram after the first extraction: After the second extraction, iron signal all along the chromatogram turned to be indistinguishable from blank, thus meaning the second extraction seems unnecessary.

#### Evaluation of inorganic iron contamination

Finally, potential contribution of the presence of inorganic iron into the protein profile was considered: two fractions of 50 µL of nasal exudate solution from a control individual were taken, to one of which 0.3 ng of free Fe^3+^ was added in order to investigate whether its addition could affect the ferroprotein profile. This spiked fraction (pH = 7.4) was incubated for 2 h at 4 °C. Then, quantitative analysis of the protein profile was accomplished by SEC-ID-ICP-MS under the conditions described in the “Experimental” section. As can be seen in Table 2, peak 3 is the sole one that clearly increases with the amount of iron added, peak that corresponds to the low molecular weight region (LMW).

**Table 2:** Integration time (min) and concentration of iron (ng mL^−1^) of nasal exudate solution in each of the peaks obtained in the chromatogram, comparing when no iron is added to the nasal exudate and after addition of 0.3 ng of inorganic Fe^3+^. For all peaks, the uncertainty was below 2%

	Peak 1 High molecular weight (HMW) fraction		Peak 2 Medium molecular weight (MMW) fraction		Peak 3 Low molecular weight (LMW) fraction	
	Integration time (min)	(ng mL^−1^)	Integration time (min)	(ng mL^−1^)	Integration time (min)	(ng mL^−1^)
No Fe addition	12.8–15.2	0.0119	15.8–17.5	0.0035	26.3–27.7	0.0015
0.3 ng Fe added	12.7–15.5	0.0107	15.9–17.6	0.0034	26.0–27.9	0.0044

### Quantification of total iron and iron-binding fractions in nasal exudate

Due to the characteristics of the sample collection method, there is a need to decipher best normalization strategy to fairly compare quantitative results of iron among different individuals. The absolute content of iron will depend on the amount of nasal exudate collected in the swab. Taking such fact into account, two different normalization approaches were considered: the mass of nasal exudate collected in the swab (weighing the swab before and after sample collection) and the amount of total protein. The first one avoids additional chemical work, but requires an analytical balance and data registration at the collection point. However, the measurement of total protein by BCA determination allows for a faster sample collection, avoiding the need for a balance that could not be used, for instance, in decentralized analysis. In addition, the aqueous content of the collected sample might be an inter- and intra-personal variable that would affect the sample weight but it will not affect the BCA results.

**Fig. 4:**
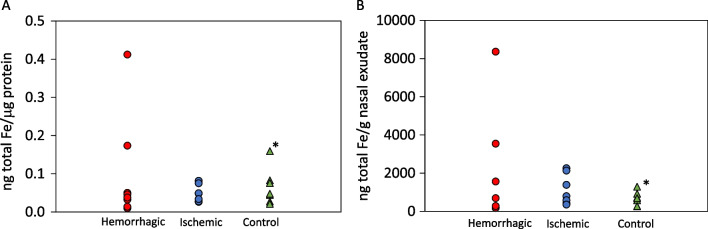
Nanograms of total iron (blank subtracted) determined for the three cohorts normalized to total BCA protein in µg (**A**) and to the mass of nasal exudate (**B**). The results marked with an asterisk * correspond to a control individual who showed a slightly higher ratio of ng Fe per µg BCA with respect to ng Fe per g of nasal exudate

Figure 4 collects the amount of total iron in ng (blank subtracted) determined for the three cohorts with respect to the total protein content determined by BCA (Fig. 4A) or normalized to the mass of nasal exudate (Fig. 4B). A similar distribution of the data is obtained for the three groups for both normalization approaches, except for a control individual (marked with an asterisk * in the figure) showing a higher ratio of ng Fe per µg BCA with respect to ng Fe per g of nasal exudate. No statistical differences have been here obtained for the evaluated samples when comparing iron concentrations of nasal exudate between hemorrhagic and ischemic groups (*p* = 0.954 and *p* = 0.524, Mann–Whitney for ng Fe normalized to BCA and normalized to g of nasal exudate, respectively), probably due to the limited number of samples available for analysis. Still, and similarly to a previous work [12], it can be observed that increased iron levels were obtained in two hemorrhagic patients, a fact that could be indicative of the severity of the stroke [20]. The control group (not investigated in the above-mentioned previous work) showed similar levels as compared to both stroke populations.

**Fig. 5:**
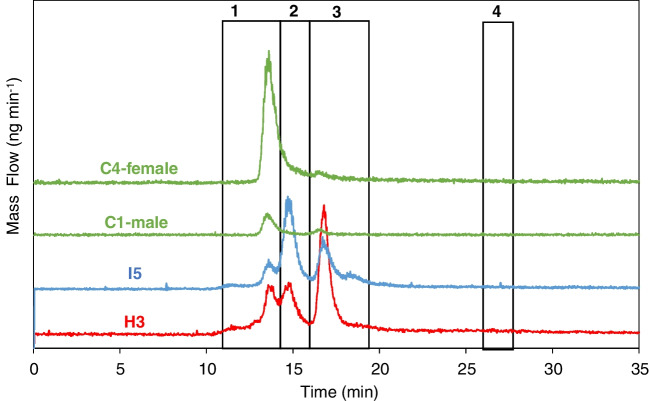
Mass flow chromatograms (ng of Fe ∙ min^−1^) with respect to retention time (min) obtained by SEC-ID-ICP-MS for a hemorrhagic (H3), ischemic (I5), and two control individuals (C1 and C4), male and female respectively. For a better visualization of the chromatographic information, baselines have been vertically displaced

Next, iron speciation profile was investigated by means of SEC-ID-ICP-MS to study potential differences between the three cohorts. Figure 5 collects a comparison of mass flow chromatograms (ng min^−1^) obtained for a hemorrhagic (H3), an ischemic (I5), and two control individuals (C1 and C4), male and female respectively. First, it can be seen that no differences in the peak profile were apparent as a function of sex. Up to three main chromatographic peaks were observed (for H3 and I5, there is also a slight increase of the signal at about 11 min, not present in all stroke individuals). Iron was not detected in the LMW region for the individuals shown in Fig. 5. Such LMW peak was only detected for two individuals within the ischemic group (IS1, IS7) among all investigated samples. Chromatographic information for the nasal exudate appears in between 10- and 20-min elution time, corresponding to the high molecular weight, HMW (600–400 kDa) and medium molecular weight MMW (400–50 kDa), being the first peak of the chromatogram eluted at a retention time of the ferritin (440 kDa) according to the column calibration.

On the sight of these results, integration of peaks was performed according to the limits shown in Fig. 5. After baseline subtraction, ng of iron bound to proteins has been calculated by integration of each of the selected areas and for each individual included within the study (see Table S2 ESI). Here, it must be highlighted the higher concentration of iron observed in the HMW fraction for hemorrhagic patients, which could be attributed to a higher concentration of HMW proteins, to a higher iron saturation of such proteins (the instrument measures iron bound to proteins), or to both. Results collected in Table S2 were also used to estimate recoveries when comparing total Fe and Fe bound to proteins, resulting in an average recovery of 99% ± 22%.

**Fig. 6:**
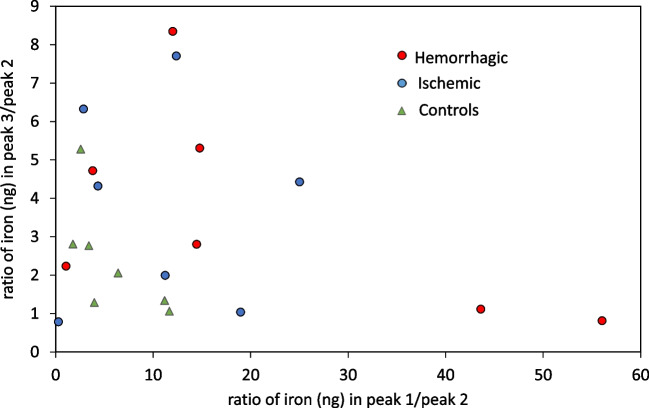
Representation of the quantitative information (ng iron) obtained after integration of the chromatographic peaks found by SEC-ID-ICP-MS and displayed in Table S2 for the different cohorts. It is shown the ratio between the ng of iron bounded to protein Peak 3 with respect to Peak 2 vs the ratio between the ng of iron bounded to protein in Peak 1 with respect to Peak 2

Regarding the MMW region, taking into account the lack for resolving proteins bound to iron in that region (transferrin 80 kDa, lactoferrin 78 kDa, and ferroportin 62.5 kDa) and according to the different chromatograms obtained, two different sections within this region were chosen to be integrated to study whether changes in the chromatograms could be distinguished between the three cohorts. The representation of the ratio between the ng of iron bound to proteins for selected peaks (see Fig. 6) has permitted to observe that control individuals show a lower data distribution variability as compared to both stroke groups.

### Concentration of selected iron-binding proteins in nasal exudate using commercial ELISA kits

**Fig. 7:**
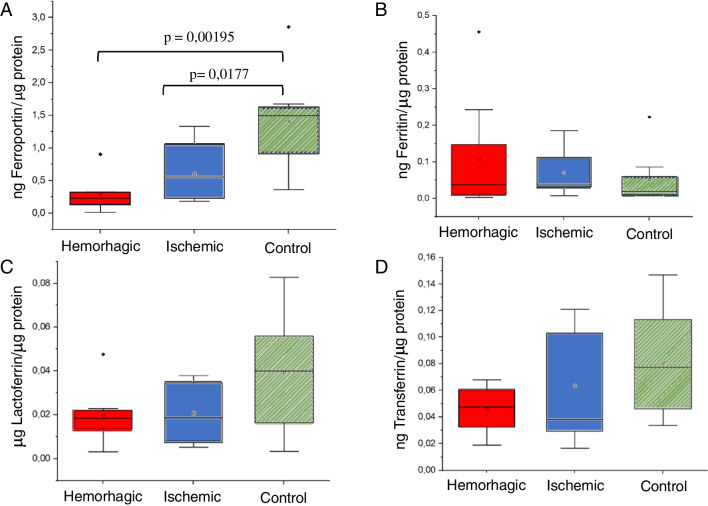
Concentration ratios with respect to BCA obtained for (**A**) ferroportin, **B** ferritin, **C** lactoferrin, and **D** transferrin, in the investigated samples obtained by means of commercial ELISA kits

Despite that different chromatographic profiles in the nasal exudate for the different cohorts were observed, it is not possible to distinguish between transferrin, lactoferrin, and ferroportin in the SEC chromatograms. Therefore, it was decided to determine the concentrations of these proteins (together with ferritin) in the nasal exudate using commercial ELISA kits, to check whether total concentration of such proteins could differ among the different cohorts. Figure 7 collects concentration of each investigated protein normalized to total protein content (statistical differences were not found between women and men in the control group for any of the investigated proteins). Data distribution, as assessed with Shapiro–Wilk’s test, showed a normal distribution for almost all investigated proteins with few exceptions: concentration of ferroportin in the case of hemorrhagic group (*p* = 0.0276), and concentration of ferritin both in the case of hemorrhagic (*p* = 0.00199) and control groups (*p* = 0.00236).

Significant differences were found for ferroportin (*p* = 0.00195, Kruskal–Wallis) between the three cohorts (Fig. 7A). Indeed, the control group showed significantly higher ferroportin levels when compared to the hemorrhagic group (*p* = 0.00195, Mann–Whitney) and also to the ischemic group (*p* = 0.0177, Mann–Whitney). It is important to highlight the drastic decrease observed for this membrane protein in hemorrhagic patients as well as in ischemic patients. Interestingly, it has been reported that the expression of ferroportin is downregulated in certain parts of the brain; the cerebral cortex and hippocampus after ischemia [22], same observation here obtained in nasal exudate. On the other hand, despite that a higher ferroportin/total protein ratio can be observed in Fig. 7A for the ischemic group as compared to the hemorrhagic group, such differences turned out not significantly different (*p* = 0.1182, Mann–Whitney).

Despite that no statistical differences were observed for ferritin in Fig. 7B, it can be clearly seen that the control group exhibits a narrower distribution along with lower average concentrations as compared to the stroke groups. It can be seen that ferritin and ferroportin concentrations show an antagonistic behavior: the higher the ferritin, the lower the ferroportin. Levels of ferritin have also showed to be increased in an animal model after induction of a hemorrhage via intra-striatal infusion of 0.5 U of type IV collagenase [26]. Moreover, it has been reported that the increase in ferritin levels after ischemia is related with a decrease in ferroportin in certain brain regions, thus leading to accumulation of iron [27]. Figure S1 in the ESM collects similar plots but normalized to the mass of nasal exudate extracted for these two proteins to further confirm the similar data distributions for both normalization strategies. Finally, Fig. 7C and D collect results for transferrin and lactoferrin in nasal exudate (with respect to total protein), for which clear trends cannot be observed, mostly attributed to the fact of the high variability for the control groups.

**Fig. 8:**
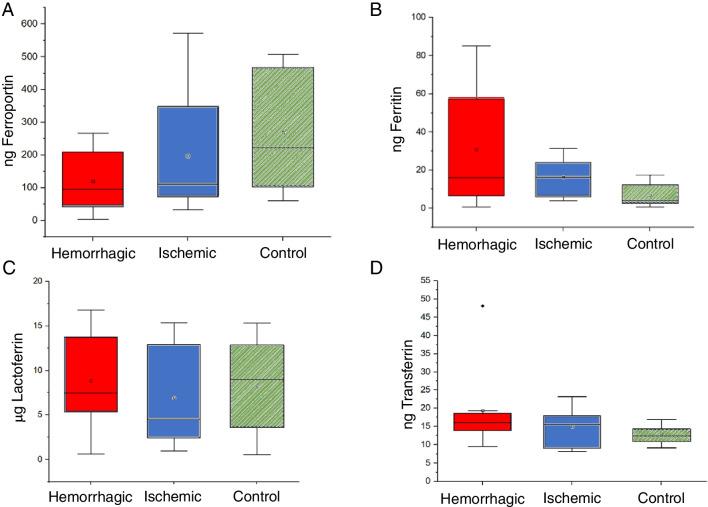
Mass obtained for (**A**) ferroportin, **B** ferritin, **C** lactoferrin, and **D** transferrin, in the investigated samples obtained by means of commercial ELISA kits

The need for a normalization strategy has been fairly justified by the type of sample collection employed. However, it would be interesting as well to evaluate whether the absolute concentration of selected Fe proteins varies among the three cohorts. For this reason, ng of ferritin, transferrin, lactoferrin, and ferroportin without any normalization approach is collected in Fig. 8. No statistical differences between the three cohorts were detected; however, results obtained for ferritin and ferroportin when comparing the three cohorts show similar distinctive trends than collected in Fig. 7. Interestingly, if the concentration ratios ferroportin/ferritin are considered, statistical differences between the control and hemorrhagic groups are obtained (*p* = 0.046, Mann–Whitney). Just statistical differences at 85% confidence level (*p* = 0.11821, *t*-test) were observed when comparing the control and ischemic groups. Finally, no statistical differences were found neither comparing the ischemic and hemorrhagic populations (*p* = 0.99354, Mann–Whitney).

Presence of proteins has been rarely investigated in nasal exudate [13, 28]. Casado et al. identified up to 111 proteins in human nasal lavage fluid of healthy subjects after precipitation of high molecular weight proteins [28]. Among Fe proteins, these authors reported the presence of transferrin, hemoglobin, and lactoferrin. For this last one, produced in nasal submucosal glands, concentration in nasal excretions has been reported to be at about 1 µg/mL [11] (nasal lavage was collected in 4 mL saline solution [29]). If lactoferrin values obtained in this work are expressed as reported by Raphael et al. [29], a concentration range between 0.2 and 3.8 µg/mL for control individuals is obtained, that is, they are in well agreement with such previous work. Lactoferrin concentration is associated with allergic processes, typically inducing an increase in the expression levels of the protein [11, 30]. It can also inhibit the growth of fibroblasts derived from nasal polyps [31]. To the best of the authors’ knowledge, no information regarding the presence of ferroportin and ferritin has been previously reported in nasal exudate. Concentration levels for these iron-related proteins and transferrin are reported here for the first time in nasal exudate both for control individuals and stroke-diagnosed individuals.

## Conclusions

In this work, we have deepen into the knowledge of the role of iron and its potential for stroke diagnosis (hemorrhagic, ischemic, and control individuals) using nasal exudate samples, as this biological fluid might allow to obtain direct clinical information from the brain thanks to the lymphatic drainage between this organ and the nasal mucosa. We demonstrated that nasal exudate can be extracted from the swab in a single step using 300 µL of 10 mM Tris/HCl, pH = 7.4. No differences in terms of data distribution were obtained neither normalizing with the mass of nasal exudate nor with the total protein concentration; however, last approach may be advantageous from the practical point of view as it does not require in situ data registration and so could be used for decentralized analyses.

Although the number of investigated samples was limited, it can be concluded that both ICP-MS speciation studies and the results for the concentrations of ferritin and ferroportin with ELISA tests showed a differential behavior between the different cohorts. Here, it should not be obviated that SEC-ID-ICP-MS did quantify Fe bounded to species and cannot be directly compared to the concentrations of proteins measured by the commercial ELISA kits as the amount of Fe bounded to such proteins might be affected by the metabolic state. In this sense, it is also important to keep in mind potential limitations of quantitative analysis given by ELISA kits, such as the risk of lack of specificity and large measurement uncertainties.

Finally, it must be highlighted that considering that the analysis of fluids requiring non-invasive sampling is a today’s trend, we believe that the studies carried out open the way for further reliable analytical applications of nasal exudate.

## Supplementary Information

Below is the link to the electronic supplementary material.

Supplementary file1 (DOCX 102 KB)

## Data Availability

All data generated or analyzed during this study are included in this article and supplementary information files.
