# Supplementary material for: Nasal exudate for diagnosis of stroke: fundamental studies through iron fractionation, total iron, and targeted protein determinations
Source: Anal Bioanal Chem. 2024 Aug 17;416(24):5365–75. doi: 10.1007/s00216-024-05469-5 (PMC11416360; doi:10.1007/s00216-024-05469-5)

**Electronic Supplementary Material**

**Nasal exudate for diagnosis of stroke: fundamental studies through iron fractionation, total iron and targeted protein determinations**

Marta Marina-Latorre^1^, Lara Lobo^1*^, Carmen García-Cabo^2^, Lorena Benavente-Fernández^2^, Sergio Calleja-Puerta^2^, M. Teresa Fernández-Abedul^1^, Héctor González-Iglesias^3^, and Rosario Pereiro^1^

*^1^Department of Physical and Analytical Chemistry, University of Oviedo, Julián Clavería 8, 33006 Oviedo (Asturias), Spain*

*^2^Department of Neurology, Central University Hospital of Asturias, Av. Roma s/n,33011 Oviedo, Spain*

*^3^Department of Technology and Biotechnology of Dairy Products, Instituto de Productos Lácteos de Asturias, Consejo Superior de Investigaciones Científicas, Spain*

Corresponding author: [lobolara@uniovi.es](mailto:lobolara@uniovi.es)

**Table S1:** Information of nasal exudate samples included in this work.

|  | **Hemorrhagic**  **(n = 8)** | | **Ischemic**  **(n = 7)** | | **Controls**  **(n = 8)** | |
| --- | --- | --- | --- | --- | --- | --- |
| Age | 71 ± 7 | | 77 ± 10 | | 64 ± 3 | |
| Sex | Males | | Males | | 50 % males | |
|  | | | | | | |
|  | **Hemorrhagic**  **(n = 8)** | | **Ischemic**  **(n = 7)** | | **Controls**  **(n = 8)** | |
| Nasal exudate weight (g) | **H1** | 0.0374 | **IS1** | 0.0388 | **C1** | 0.0146 |
|  | **H2** | 0.0242 | **IS2** | 0.0149 | **C2** | 0.0257 |
|  | **H3** | 0.0375 | **IS3** | 0.0045 | **C3** | 0.0223 |
|  | **H4** | 0.0406 | **IS4** | 0.0146 | **C4** | 0.0296 |
|  | **H5** | 0.0092 | **IS5** | 0.0376 | **C5** | 0.0129 |
|  | **H6** | 0.0149 | **IS6** | 0.0039 | **C6** | 0.0092 |
|  | **H7** | 0.0143 | **IS7** | 0.0256 | **C7** | 0.0224 |
|  | **H8** | 0.1084 |  |  | **C8** | 0.0183 |

|  | **Speciation of Fe-binding proteins (ng)** | | | |
| --- | --- | --- | --- | --- |
|  | HMW | MMW | | LMW |
|  | Peak 1  (10.5-15.2) min | Peak 2  (15.3-16.1) min | Peak 3  (16.1-19.2) min | Peak 4  (24.7-25.6) min |
| **H1** | 0.795 | 0.210 | 0.992 | -- |
| **H2** | 0.720 | 0.060 | 0.501 | -- |
| **H3** | 1.057 | 1.007 | 2.253 | -- |
| **H5** | 16.362 | 0.292 | 0.239 | -- |
| **H6** | 8.893 | 0.204 | 0.228 | -- |
| **H7** | 0.945 | 0.064 | 0.34 | -- |
| **H8** | 1.574 | 0.109 | 0.306 | -- |
| **IS1** | 3.526 | 0.141 | 0.625 | 0.052 |
| **IS2** | 2.345 | 0.209 | 0.418 | -- |
| **IS3** | 1.820 | 0.096 | 0.1 | -- |
| **IS4** | 0.766 | 0.062 | 0.478 | -- |
| **IS5** | 0.434 | 1.595 | 1.262 | -- |
| **IS6** | 0.147 | 0.034 | 0.147 | -- |
| **IS7** | 0.346 | 0.122 | 0.772 | 0.260 |
| **C1** | 0.320 | 0.050 | 0.103 | -- |
| **C2** | 0.136 | 0.053 | 0.28 | -- |
| **C3** | 2.179 | 0.187 | 0.199 | -- |
| **C4** | 2.234 | 0.200 | 0.268 | -- |
| **C5** | 0.177 | 0.052 | 0.144 | -- |
| **C6** | 0.037 | 0.021 | 0.059 | -- |
| **C8** | 0.598 | 0.151 | 0.195 | -- |

**Table S2:** Nanograms of Fe obtained after integration of the chromatogrpahic peaks and its corresponding integration times for all investigated samples.

**Figure S1:** Concentration ratios of Ferroportin (A) and Ferritin (B) respect to the mass of nasal exudate collected (in ng/g) for each sample.


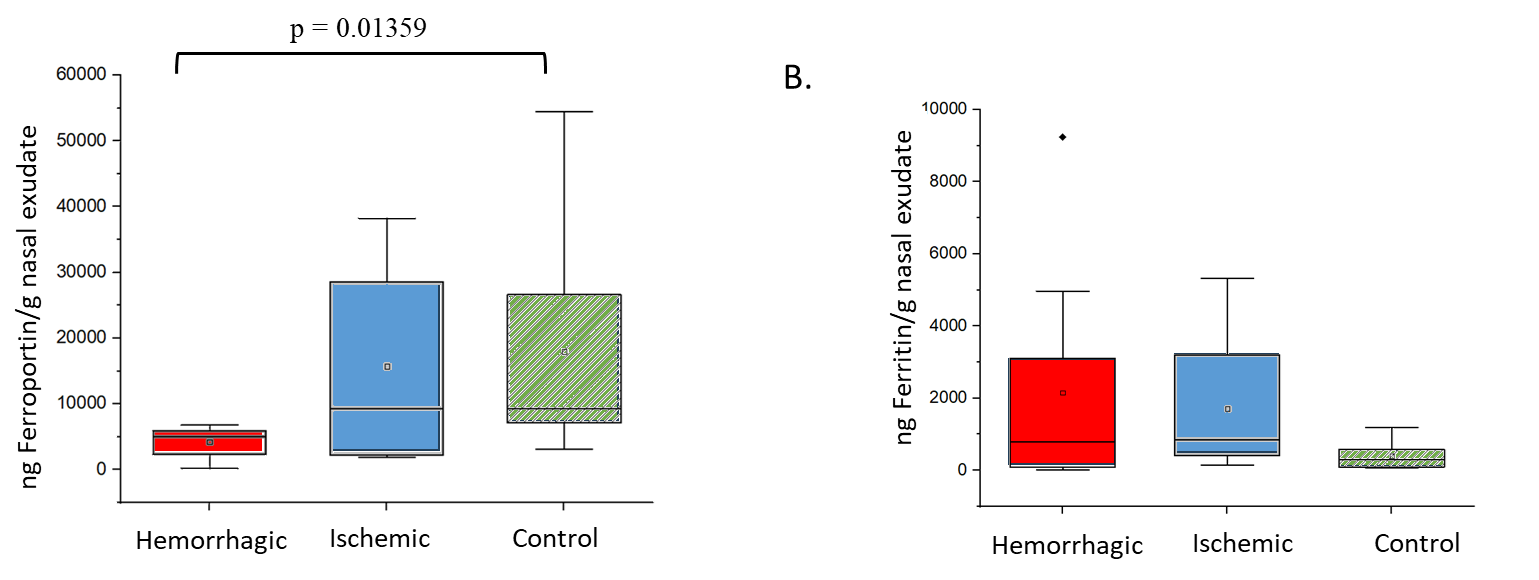

Supplement: Supplementary file 1 — Supplementary file1 (DOCX 102 KB) [file 216_2024_5469_MOESM1_ESM.docx]
